# Extraction of Proanthocyanidins from Chinese Wild Rice (*Zizania latifolia*) and Analyses of Structural Composition and Potential Bioactivities of Different Fractions

**DOI:** 10.3390/molecules24091681

**Published:** 2019-04-30

**Authors:** Mei-Jun Chu, Yong-Mei Du, Xin-Min Liu, Ning Yan, Feng-Zhong Wang, Zhong-Feng Zhang

**Affiliations:** 1 Tobacco Research Institute, Chinese Academy of Agricultural Sciences, Qingdao 266101, China; chumjun@163.com (M.-J.C.); duyongmei@caas.cn (Y.-M.D.); liuxinmin@caas.cn (X.-M.L.); yanning@caas.cn (N.Y.); 2 Institute of Food Science and Technology, Chinese Academy of Agricultural Sciences, Beijing 100193, China; wangfengzhong@caas.cn

**Keywords:** wild rice, *Zizania latifolia*, proanthocyanidins, extraction, purification, fractionation, degree of polymerization, bioactivities

## Abstract

Due to the importance of proanthocyanidin bioactivity and its relationship with chemical structure, ultrasound-assisted extraction and purification schemes were proposed to evaluate the proanthocyanidin content and analyze the structural composition and potential bioactivities of different proanthocyanidin fractions from Chinese wild rice (*Zizania* *latifolia*). Following an optimized extraction procedure, the crude wild rice proanthocyanidins (WRPs) were purified using n-butanol extraction, chromatography on macroporous resins, and further fractionation on Sephadex LH-20 to yield six specific fractions (WRPs-1–WRPs-6) containing proanthocyanidin levels exceeding 524.19 ± 3.56 mg/g extract. Structurally, (+)-catechin, (−)-epicatechin, and (−)-epigallocatechin were present as both terminal and extension units, and (−)-epicatechin was the major extension unit, in each fraction. This is the first preparation of WRP fractions with a different mean degree of polymerization (mDP), ranging from 2.66 ± 0.04 to 10.30 ± 0.46. A comparison of the bioactivities of these fractions revealed that fractions WRPs-1−WRPs-5 had significant DPPH radical scavenging activities, whereas fraction WRPs-6 with a high mDP showed better α-glucosidase and pancreatic lipase inhibitory effects. These findings should help define possible applications of WRPs to functional foods or nutraceuticals.

## 1. Introduction

Wild rice, the seed of the aquatic plant *Zizania* (family Poaceae), has long been recognized as a health-promoting whole grain, particularly because of its health benefits, including the ability to suppress oxidative stress, reduce hyperlipidemia, and prevent atherogenesis, type 2 diabetes, and obesity [1,2,3]. Since wild rice is gluten-free and safe for human consumption, the growing interest in wild rice has increased its commercialization in North America [4]. There are four known species of wild rice. Of these, *Z. aquatica*, *Z. palustris*, and *Z. texana* are indigenous to North America, whereas *Z. latifolia* is native to China, Japan, and Vietnam [5]. Chinese wild rice (*Z. latifolia*), which is widely distributed in areas along the Yangtze and Huai Rivers, is an age-old food that has been traditionally used to treat a variety of ailments in Chinese medicinal practice [2,6]. Wild rice is a rich source of phenolic compounds including phenolic acids, flavonoid glycosides, and proanthocyanidins. These compounds are the key contributors to the health benefits of wild rice [4,7,8].

Proanthocyanidins, commonly known as condensed tannins, are secondary metabolites that are found in a wide variety of plant-based foods including fruits, vegetables, and grains (e.g., grape, cranberry, litchi, cinnamon, cocoa, and peanut), particularly in the skin and seeds [9,10,11]. Proanthocyanidins are a class of phenolic oligomers and polymers composed of flavan-3-ol monomer units. The constitutive units are linked through a single bond between C-4 of the upper unit and C-6 or C-8 of the lower unit (B-type), which can coexist with an additional bond between adjacent flavan-3-ol units that connects C-2 of the upper unit via an oxygen atom to C-7 or C-5 of the lower unit (A-type) [10]. In recent years, the demand for proanthocyanidins has increased [9], since proanthocyanidin-rich diets are associated with a reduced risk of chronic cardiovascular diseases, including hypertension and dyslipidemia [12]. Additionally, proanthocyanidins derived from other plants have been reported to have remarkable in vitro antioxidant activity, as well as inhibitory effects on α-glucosidase and pancreatic lipase [9,13,14,15]. α-Glucosidase is a key enzyme in the digestion of complex carbohydrates, whereas pancreatic lipase inhibitors can suppress triglyceride absorption, leading to potential antiobesity effects. Inhibition of these two enzymes is, therefore, a valid strategy in managing blood glucose level and obesity [16]. We previously revealed a high content of proanthocyanidins in Chinese wild rice [7]. However, in-depth studies on the extraction, purification, and fractionation of wild rice proanthocyanidins (WRPs) have not been reported. The subunit composition, mean degree of polymerization (mDP), and the related antioxidant, antidiabetic, and antiobesity activities of WRPs remain unknown.

The present study aimed to investigate the chemical structure, including subunit composition and mDP, and potential bioactivities of different WRP fractions. For this purpose, crude WRPs were obtained under optimal extraction conditions achieved by response surface methodology (RSM). The active n-butanol fraction was purified and then fractionated by Sephadex LH-20 chromatography to yield several distinct WRP fractions. After acid-catalysis in the presence of phloroglucinol, these isolated fractions were analyzed by ultra-performance liquid chromatography (UPLC) coupled with linear ion trap quadrupole-Orbitrap-mass spectrometry (LTQ-Orbitrap-MS) to provide structural information of the WRPs. Furthermore, the potential antioxidant, antidiabetic, and antiobesity activities as assessed by the in vitro inhibitory effects on DPPH radical, α-glycosidase, and pancreatic lipase of the different fractions were examined.

## 2. Results and Discussion

### 2.1. Extraction of WRPs

#### 2.1.1. Single Factor Experimental Analysis

Since extraction is the most important step to recover the highest amount of the target compounds from plants [17], a preliminary single factor experiment was conducted to determine the effects of operation parameters of ultrasound-assisted extraction (UAE) on the content of WRPs. According to the experimental results (Figure S1 in the Supplementary Materials), the four most important independent variables at three levels, namely the concentration of aqueous ethanol (80, 90, and 100%; *v*/*v*), the liquid-solid ratio (40, 50, and 60 mL/g; *v*/*w*), the extraction temperature (30, 40, and 50 °C), and the ultrasonic power (300, 350, and 400 W), were selected for optimizing the extraction conditions using RSM based on a Box–Behnken design (BBD).

#### 2.1.2. Fitting the Model

The corresponding experimental results for the content of WRPs of each run in BBD are presented in Table S1 in the Supplementary Materials. By applying multiple regression analysis on the experimental data, the equation describing the correlation between WRPs content and the four variables was as follows:

Y = 6.09 + 0.80X_1_ − 0.10X_2_ − 0.098X_3_ − 0.075X_4_ − 0.014X_1_X_2_ − 0.30X_1_X_3_ − 0.14X_1_X_4_ − 0.043X_2_X_3_ − 0.26X_2_X_4_ + 0.19X_3_X_4_ − 1.37X_1_^2^ − 1.19X_2_^2^ − 0.25X_3_^2^ − 0.30X_4_^2^ (1)

where Y is the WRPs content (mg/g rice); X_1_, X_2_, X_3_, and X_4_ represent the coded variables for the concentration of aqueous ethanol, the liquid-solid ratio, the extraction temperature, and the ultrasonic power, respectively.

The data for the analysis of variance (ANOVA) statistical test of the model are shown in Table 1. The small *p*-value (*p* < 0.0001) suggested that the model was highly significant. Meanwhile, a lack of fit *F*-value of 2.88 and an associated *p*-value of 0.1596 implied that there was an excellent agreement between the experimental values and the predicted values. In addition, a determination coefficient (R^2^) of 0.9941 and an adjusted determination coefficient (adj R^2^) of 0.9882 indicated that there was a satisfactory correlation between the experimental WRPs content and the values predicted by the equation. A high degree of precision and good reliability of the data were demonstrated by a low coefficient of variation (CV = 2.24%). These results suggested that the model works well at predicting WRPs extraction.

#### 2.1.3. Optimization of Extraction Conditions

**Figure 1:**
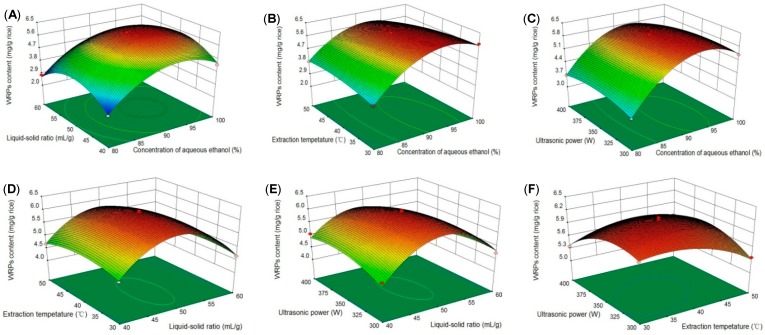
Response surface plots showing interaction effects of concentration of aqueous ethanol and liquid-solid ratio (**A**), concentration of aqueous ethanol and extraction temperature (**B**), concentration of aqueous ethanol and ultrasonic power (**C**), liquid-solid ratio and extraction temperature (**D**), liquid-solid ratio and ultrasonic power (**E**), extraction temperature and ultrasonic power (**F**) on the WRPs content.

Three-dimensional surface plots were used to explain the interactions of the variables and to determine the optimal level of each independent variable to produce a maximal response. When the extraction temperature and ultrasonic power were set at zero level, the WRPs content increased initially as the concentration of aqueous ethanol and the liquid-solid ratio increased and then slightly decreased (Figure 1A). Similar effects on WRPs content of other variables are shown in Figure 1B–F. The results were in accordance with the single factor experiment and the ANOVA analysis. The maximum predicted value of WRPs content (6.28 mg/g rice) could be obtained under the following optimal conditions: a concentration of aqueous ethanol of 93.73%, a solid-liquid ratio of 50.07 mL/g, an extraction temperature of 44.39 °C, and an ultrasonic power of 330.34 W. Considering the operability in actual processing procedures, a verification experiment was carried out under the following modified conditions: a concentration of aqueous ethanol of 94%, a solid-liquid ratio of 50 mL/g, an extraction temperature of 44 °C, and an ultrasonic power of 330 W. Under these conditions, the WRPs content of the extract from the verification experiment was 6.33 ± 0.10 mg/g rice, which was in good agreement with the predicted value.

### 2.2. Proanthocyanidin Content and In Vitro Bioactivities of the Isolated Fractions

**Figure 2:**
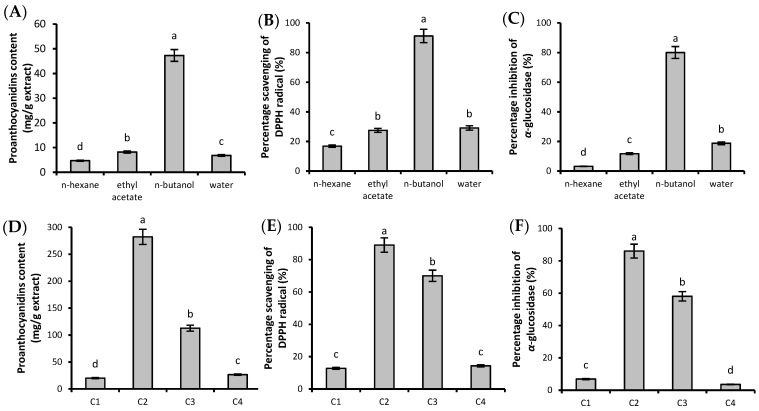
(**A**–**F**) Proanthocyanidin content, DPPH radical scavenging activity, and α-glucosidase inhibitory activity of different fractions (*c* = 0.2 mg/mL). Different letters above each bar within the same figure indicate significant differences (*p* < 0.05).

The crude WRPs obtained under the optimal UAE condition were first divided into four fractions soluble in n-hexane, ethyl acetate, n-butanol, and water, respectively. The proanthocyanidin content, DPPH radical scavenging activity, and α-glucosidase inhibitory activity were evaluated to determine the active fractions. As shown in Figure 2A–C, the n-butanol fraction had the highest proanthocyanidin content, DPPH radical scavenging activity, and α-glucosidase inhibitory activity.

The active n-butanol fraction was then further loaded onto a D101 macroporous adsorption resin column to obtain four fractions (fractions C1–C4), which were screened for their proanthocyanidin content, antioxidant activity, as well as α-glucosidase and pancreatic lipase inhibitory activities. As shown in Figure 2D–F, the fractions C2 and C3, with the highest proanthocyanidin contents of 282.24 ± 2.07 and 112.80 ± 1.55 mg/g extract, respectively, displayed good DPPH radical scavenging activity and α-glucosidase inhibitory activities. The proanthocyanidins were, therefore, mainly distributed in fractions C2 (10–30% ethanol effluents) and C3 (40–60% ethanol effluents), which were combined and concentrated for next fractionation. All the four fractions at a concentration of 10 mg/mL showed no obvious inhibitory activity against pancreatic lipase.

To further study the structure and in vitro bioactivities of WRPs, the proanthocyanidin-rich fractions (fractions C2 and C3) were fractionated on a Sephadex LH-20 column to obtain six fractions (fractions WRPs-1–WRPs-6), which were measured for the proanthocyanidin content, DPPH radical scavenging activity, as well as α-glucosidase and pancreatic lipase inhibitory activities. As can be seen in Table 2, all the isolated fractions had high proanthocyanidin contents, exceeding 524.19 ± 3.56 mg/g extract, with fraction WRPs-3 having the highest level of 863.81 ± 8.02 mg/g extract. The scavenging effects on DPPH radical were in the order: WRPs-3 ≈ WRPs-4 ≈ WRPs-5 > WRPs-2 > WRPs-1 > ascorbic acid > WRPs-6. For α-glucosidase inhibitory activities of the six fractions, the two fractions WRPs-5 and WRPs-6 exhibited excellent inhibitory effect with IC_50_ values of 117.72 ± 1.45 and 84.01 ± 0.74 μg/mL, respectively, lower than that of acarbose (IC_50_ = 186.31 ± 1.04 μg/mL). In the pancreatic lipase assay, fractions WRPs-1–WRPs-5 displayed no obvious inhibitory activities (IC_50_ > 2000 μg/mL), whereas fraction WRPs-6 showed a weak inhibitory effect on pancreatic lipase (IC_50_ = 1054.01 ± 6.67 μg/mL).

These data suggested that the fractions WRPs-1–WRPs-5 had significant DPPH radical scavenging abilities, whereas fraction WRPs-6 showed potent α-glucosidase and mild pancreatic lipase inhibitory activities. The present fractionation can filter out effective in vitro antioxidants (fractions WRPs-1–WRPs-5) and α-glucosidase and pancreatic lipase inhibitors (fraction WRPs-6) to some extent.

### 2.3. Structural Composition of Fractions WRPs-1–WRPs-6

**Figure 3:**
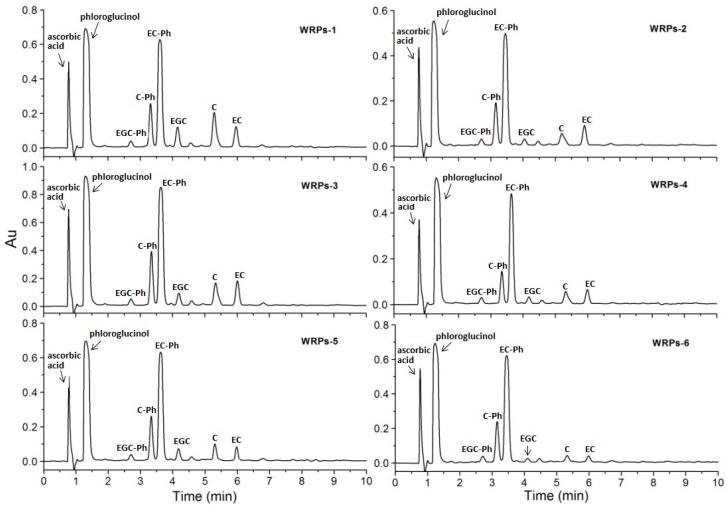
Reversed-phase UPLC chromatograms of fractions WRPs-1–WRPs-6 after phloroglucinolysis at 280 nm. Abbreviations: EGC-Ph, (−)-epigallocatechin-phloroglucinol derivative; C-Ph, (+)-catechin-phloroglucinol derivative; EC-Ph, (−)-epicatechin-phloroglucinol derivative; EGC, (−)-epigallocatechin; C, (+)-catechin; EC, (−)-epicatechin.

The reversed-phase UPLC chromatograms of fractions WRPs-1–WRPs-6 (Figure S2 in the Supplementary Materials) implied that they have distinctly different chemical compositions. The structural composition of fractions WRPs-1–WRPs-6 was determined by using phloroglucinolysis, which has been proved to be an efficient method for structure analysis of proanthocyanidins [18,19]. In the degradation reaction, terminal units of proanthocyanidins are liberated as free flavan-3-ol monomers, while the extension units, released as electrophilic intermediates, are attacked by the nucleophile (phloroglucinol) to form the corresponding phloroglucinol adducts. The composition of the flavan-3-ol units and the mDP of the proanthocyanidins were determined by UPLC-LTQ-Orbitrap-MS analysis of the cleavage products. Figure 3 shows the reversed-phase UPLC chromatograms of the fractions WRPs-1–WRPs-6 after phloroglucinolysis. The major cleavage products detected were identified by comparing their retention times and MS data with those of authentic standards or literature data [11,20], and results are listed in Table 3. The WRPs were mainly composed of (+)-catechin (C), (−)-epicatechin (EC), and (−)-epigallocatechin (EGC) as terminal and extension units. The proportions of constitutive units and mDP of proanthocyanidins in each fraction were also calculated (Table 4). The terminal units of fractions WRPs-1–WRPs-6 were mainly C and EC with proportions of 4.21 ± 0.12–15.15 ± 0.30% and 3.80 ± 0.06–11.83 ± 0.42%, respectively. With respect to the extension units, EC with increased contents, from fractions WRPs-1 to WRPs-6, had the highest proportions (44.03 ± 0.53–70.63 ± 0.69%), followed by C (15.79 ± 0.21–22.89 ± 0.26%), suggesting EC to be the major extension unit of WRPs. Compared with C and EC, EGC was found at much lower levels in the terminal and extension units (1.70 ± 0.04–9.55 ± 0.33% and 2.87 ± 0.08–3.87 ± 0.10%, respectively). The mDP gradually increased from fractions WRPs-1 to WRPs-6, with fraction WRPs-1 having a minimum of 2.66 ± 0.04 and fraction WRPs-6 having a maximum of 10.30 ± 0.46.

Upon comparing the aforementioned information with the bioactivities of fractions WRPs-1–WRPs-6, it is clear that the structure of WRPs greatly affects their bioactivity. The DPPH radical scavenging activity was the lowest in fraction WRPs-6 with the largest mDP, followed by fraction WRPs-1 with the smallest mDP, indicating that there was an increase and then a fall in DPPH radical scavenging activity as the mDP increased. Similarly, Jerez et al. found that for proanthocyanidins from the bark of *Pinus radiata*, there was an increase in DPPH radical scavenging activity up to 6.5 mDP and then a fall in DPPH scavenging activity as mDP further increased (9.2–14.6 mDP) [21]. With respect to enzyme inhibitory activities, fraction WRPs-6, which had the largest mDP, showed the highest inhibitory activities against α-glucosidase and pancreatic lipase. Previous reports also suggested that the α-glucosidase inhibitory activity of proanthocyanidins from the leaves of *Chamaecyparis obtusa* var. *formosana* and pancreatic lipase inhibitory activity of proanthocyanidins from the fruits of *Diospyros kaki* both increased with mDP [22]. Therefore the mDP of WRPs is a significant determinant of their promising potential to be used as natural food antioxidants and enzyme inhibitors.

## 3. Materials and Methods

### 3.1. Plant Materials and Chemicals

Wild rice *Z. latifolia* was collected from Jiangling County, Jingzhou City, Hubei Province, China (30°13′10″ N; 112°34′5″ E), in September 2017. The sample was obtained by manually harvesting mature plant tassels, drying and then dehulling them to obtain the seeds. The seeds were ground to a fine powder in a mechanical grinder and then sieved through a 0.45 mm sifter.

(+)-Catechin (C), (−)-epicatechin (EC), and (−)-epigallocatechin (EGC), DPPH (2,2-diphenyl-1-picrylhydrazyl), α-glucosidase (type I, from Saccharomyces cerevisiae), and pancreatic lipase (type II, from porcine pancreas) were purchased from Sigma-Aldrich Chemical Co., (St. Louis, MO, USA). Ascorbic acid, acarbose, and orlistat were obtained from Macklin Biochemical Co., Ltd. (Shanghai, China). Phloroglucinol was from Aladdin Reagents Co., Ltd. (Shanghai, China). HPLC and LC–MS grade solvents were bought from Sinopharm Chemical Reagent Co., Ltd. (Shanghai, China). The D101 macroporous resin and Sephadex LH-20 were from Solarbio Science & Technology Co., Ltd. (Beijing, China) and GE Healthcare Bio-Sciences AB (Uppsala, Sweden), respectively. Precoated silica gel plates (GF254, Qingdao Marine Chemical Co., Ltd., Qingdao, China) were used for thin layer chromatography (TLC) analysis, and spots were visualized by spraying with anisaldehyde-sulfuric acid agent.

### 3.2. Experimental Design to Set Up the Proanthocyanidins Extraction

#### 3.2.1. Ultrasound-Assisted Extraction Procedure

The WRPs were extracted using a KQ-2200DB ultrasonic cleaning bath (Kunshan Co., Ltd., Kunshan, China) and the temperature was preset before the extraction process. After extraction, the mixture was centrifuged at 3000× *g* for 20 min, and the combined supernatants were used as the crude WRPs to determine the proanthocyanidin content.

#### 3.2.2. Experimental Design

According to previous studies [23,24], a single factor experiment was firstly performed to investigate the effects of the concentration of aqueous ethanol (60, 70, 80, 90, and 100%; EtOH %, *v*/*v*), the liquid-solid ratio (20, 30, 40, 50, and 60 mL/g; *v*/*w*), the extraction temperature (30, 40, 50, 60, and 70 °C), the ultrasonic power (200, 250, 300, 350, and 400 W), and the extraction time (20,30, 40, 50, and 60 min) on the content of WRPs obtained. Subsequently, a BBD with four important variables at three levels, which were determined based on the single factor experimental results, was employed to optimize the extraction conditions. The combined effects of the four independent variables: the concentration of aqueous ethanol (EtOH %, X_1_), the liquid-solid ratio (mL/g, X_2_), the extraction temperature (°C, X_3_), and the ultrasonic power (W, X_4_) were evaluated. The coded and uncoded (actual) levels of the independent variables are listed in Table S1 in the Supplementary Materials. The response variables were fitted to the following quadratic polynomial model:

(2) $$Y=\beta_{0}+\overset{4}{\underset{i=1}{\sum }}\beta_{i}X_{i}+\overset{4}{\underset{i=1}{\sum }}\beta_{\mathrm{ii}}X_{i}^{2}+\overset{3}{\underset{i=1}{\sum }}\overset{4}{\underset{j=i+1}{\sum }}\beta_{\mathrm{ij}}X_{i}X_{j}$$

where Y represents the response variable, WRPs content, X_i_ and X_j_ are the independent variables affecting the response, and β_0_, β_i_, β_ii_, and β_ij_ are the regression coefficients of the model (intercept, linear, quadratic, and interaction terms, respectively).

The Design Expert Software (Version 8.0.6; Stat-Ease Inc., Minneapolis, MN, USA) was used for the experimental plan, data analysis, model generation, and determination of optimum conditions. The relationship between independent variables and responses was analyzed by response surface plots. Optimum conditions for UAE were calculated according to the desirability function.

### 3.3. Partition and Purification of Crude WRPs

Using a previously described method for preparing proanthocyanidins [25], the crude WRPs obtained by UAE were successively partitioned with n-hexane, ethyl acetate, n-butanol, and water (three times for each) to yield soluble fractions of n-hexane (yield 18.17%, *w*/*w*), ethyl acetate (yield 11.62%), n-butanol (yield 26.08%), and water (yield 44.13%), respectively. The n-butanol soluble fraction was then subjected to a D101 macroporous adsorption resin column (16 × 300 mm), eluting with deionized water (100 mL), and 10%, 20%, 30%, 40%, 50%, 60%, 70%, 80%, 90%, and 100% aqueous ethanol solution (EtOH%, *v*/*v*, 250 mL for each, 2 mL/min). The effluents were collected sequentially and then combined to obtain four fractions (fractions C1–C4) based on a TLC analysis. In general, the water eluent was collected as fraction C1 (yield 33.51%, *w*/*w*). The 10–30% ethanol eluents were combined as fraction C2 (yield 20.35%). The eluents of 40–60% ethanol gave fraction C3 (yield 8.06%). The 70–100% ethanol eluents produced fraction C4 (yield 27.23%).

### 3.4. Fractionation of WRPs

The proanthocyanidin-rich fractions C2 and C3 were combined and then loaded onto a Sephadex LH-20 column (10 × 800 mm). The eluting solvent gradient used was based on that described by Wei et al. [26], with slight modifications. In brief, the column was washed with deionized water (150 mL), followed by methanol-water (40:60, *v*/*v*; 150 mL) to remove sugars, glycosides, and monomeric phenols [27], and then sequentially eluted with methanol-water (60:40, *v*/*v*; 100 mL), methanol-water (75:25, *v*/*v*; 100 mL), methanol-water (90:10, *v*/*v*; 100 mL), acetone-methanol-water (10:80:10, *v*/*v*; 100 mL), acetone-methanol-water (20:65:15, *v*/*v*; 50 mL), acetone-methanol-water (30:40:30, *v*/*v*; 50 mL), and finally acetone-water (70:30, *v*/*v*; 100 mL), at a flow rate of 0.15 mL/min. The effluents were collected using a fraction collector (3 mL per tube). Following a TLC analysis using methanol/ethyl acetate/isopropanol/formic acid (1:10:1:1 and 1:7:1:1, *v*/*v*) as the developing solvents, six fractions were pooled and concentrated: WRPs-1 (effluents of methanol-water 60:40 and 75:25), WRPs-2 (effluents of methanol-water 90:10), WRPs-3 (effluents of methanol-water 90:10 and acetone-methanol-water 10:80:10), WRPs-4 (effluents of acetone-methanol-water 10:80:10 and 20:65:15), WRPs-5 (effluents of acetone-methanol-water 30:40:30), and WRPs-6 (effluents of acetone-water 70:30). The yields for fractions WRPs-1–WRPs-6 were 26.43%, 21.22%, 14.30%, 6.49%, 13.55%, and 8.87% (*w*/*w*), respectively.

### 3.5. Acid-Catalysis in the Presence of Phloroglucinol (Phloroglucinolysis)

Phloroglucinolysis of the isolated fractions WRPs-1–WRPs-6 was performed based on an earlier study [18]. Briefly, 5 mg of sample was reacted in 1 mL of 0.1 N HCl in methanol containing 50 g/L phloroglucinol and 10 g/L ascorbic acid in a stoppered test tube. The reaction mixture was heated at 50 °C for 20 min, after which 5 mL of 40 mM aqueous sodium acetate was added to stop the reaction. The cleavage products were immediately analyzed by UPLC-LTQ-Orbitrap-MS.

### 3.6. UPLC-LTQ-Orbitrap-MS Analysis

A Waters ACQUITY H-CLASS UPLC instrument with a photo-diode array and an autosampler (Waters, Milford, MA, USA), coupled with a linear ion trap quadrupole Orbitrap XL mass spectrometer (Thermo Scientific, San Jose, CA, USA), was used to analyze the cleavage products. For the analysis, 2 μL of sample was injected onto a Waters ACQUITY UPLC BEH C18 column (1.7 µm, 2.1 × 50 mm) and eluted with a mobile phase gradient consisting of 0.1% (*v*/*v*) acetic acid in acetonitrile (solvent A) and 0.1% (*v*/*v*) acetic acid in water (solvent B), at a flow rate of 0.3 mL/min. The solvent system was as follows: 0–4 min, 5–10% A; 4–6 min, 10–15% A; 6–8 min, 15–60% A; 8–9 min, 60–90% A; and 9–10 min, 90–95% A.

For mass detection, an electrospray ionization source was operated in positive mode with a scan range from *m*/*z* 150 to 1500. The capillary temperature was 350 °C. Nitrogen was used as the sheath gas and auxiliary gas, and the gas flow was set at 30 and 5 arbitrary units, respectively. The spray voltage was 4000 V. The collision energy was 35% to adjust for collision-induced dissociation for the best performance. The Xcalibur 2.1 software (Thermo Scientific, San Jose, CA, USA) was used for data analysis.

The identification of C, EC, EGC, and the corresponding phloroglucinol adducts was achieved by comparing their UPLC retention times and MS data with those of authentic standards or literature data. C, EC, and EGC were quantified based on their standards. For quantitation of the phloroglucinol adducts, the respective flavan-3-ol monomers were used as the standards. With this procedure, the phloroglucinol adducts were assumed to have the same molar absorptivities as their respective flavan-3-ol monomers [18,28]. To calculate the mDP of the proanthocyanidins, the sum of all subunits (flavan-3-ol monomers and phloroglucinol adducts, in moles), was divided by the sum of all flavan-3-ol monomers (in moles).

### 3.7. Determination of Proanthocyanidin Content

The proanthocyanidin content was measured using a modified vanillin-H_2_SO_4_ method [29]. Briefly, 20 μL of the sample was mixed with 100 μL of vanillin methanol solution (30 g/L) and 100 μL of H_2_SO_4_ methanol solution (30%, *v*/*v*). (+)-Catechin was used as a reference. After incubation at room temperature for 5 min in the dark, the absorbance at 500 nm was measured using a Multiskan GO microplate spectrophotometer (Thermo Scientific, San Jose, CA, USA). The results for the crude WRPs and the isolated fractions were expressed as milligram (+)-catechin equivalents per gram of rice (mg/g rice) and milligram (+)-catechin equivalents per gram of extract (mg/g extract), respectively.

### 3.8. DPPH Radical Scavenging Assay

The DPPH radical scavenging activity was evaluated using the method described by Yuan et al. [30], with ascorbic acid as the positive control. The percentage of scavenging was calculated according to the following equation:

Percentage scavenging of DPPH radical (%) = (1 − A_sample_/A_control_) × 100 (3)

where A_sample_ represents the absorbance of the DPPH solution in the presence of the sample and A_control_ is the absorbance of the DPPH solution without sample (which was replaced by the solvent). The corresponding IC_50_ value, defined as the concentration of extract required to scavenge 50% of the DPPH radicals, was also calculated, and the result was expressed as micrograms extract per milliliter solvent (µg/mL).

### 3.9. α-Glucosidase Inhibition Assay

The α-glucosidase inhibition assay was carried out according to a previously reported method [31], with some modifications. Briefly, 10 μL of the test sample in DMSO was mixed with 620 μL of phosphate buffered saline (PBS; 0.1 M, pH 6.9) after which 10 μL of α-glucosidase (2 U/mL) in PBS was added and incubated at 37 °C for 10 min. Following this, 200 μL of 6 mM *p*-nitrophenyl α-D-glucopyranoside in PBS was added and incubated at 37 °C for another 20 min. The reaction was terminated by adding 1 mL of 1 M sodium carbonate aqueous solution and the absorbance at 400 nm was measured. Acarbose was used as the positive control. The percentage inhibition was calculated as follows:

Percentage inhibition of α-glucosidase (%) = [1 − (A_sample_ − A_background_)/(A_control_ − A_background_)] × 100 (4)

where A_sample_ represents the absorbance of the test sample, A_control_ is the absorbance of the control solution without sample (which was replaced by DMSO), and A_background_ is the absorbance of the background solution without α-glucosidase (which was replaced by PBS). The IC_50_ value (µg/mL) as similarly defined for the DPPH radical was also determined.

### 3.10. Pancreatic Lipase Inhibition Assay

The pancreatic lipase activity was measured using a previous method [31], with some modifications. Briefly, 10 μL of the test sample in DMSO was added to 500 µL of Tris-HCl buffer (0.1 M, pH 8.0), after which 200 μL of pancreatic lipase (2 mg/mL) in Tris–HCl buffer was added and incubated at 37 °C for 15 min. Following this, 200 μL of 12.5 mM *p*-nitrophenyl butyrate in Tris–HCl buffer was added and incubated at 37 °C for 20 min. The absorbance at 400 nm was measured and orlistat was used as the positive control. The percentage inhibition of pancreatic lipase was calculated as follows:

Percentage inhibition of pancreatic lipase (%) = [1 − (A_sample_ − A_background_)/(A_control_ − A_background_)] × 100 (5)

where A_sample_ represents the absorbance of the test sample, A_control_ is the absorbance of the control solution without the sample (which was replaced by DMSO), and A_background_ is the absorbance of the background solution without pancreatic lipase (which was replaced by Tris–HCl). The IC_50_ value (µg/mL) was calculated.

### 3.11. Statistical Analysis

All results are the averages of at least three assay replicates and are expressed as the mean ± standard deviation (SD). Statistical analysis was performed using the SPSS 19.0 software (SPSS Inc., Chicago, IL, USA). *p*-Values < 0.05 were considered to be statistically significant.

## 4. Conclusions

In the present study, an optimized UAE was applied to extract proanthocyanidins from Chinese wild rice with high yield. Six specific fractions with mDP in increasing order were obtained after partition, purification, and fractionation of crude WRPs. Fractions WRPs-1–WRPs-5 possessed excellent DPPH radical scavenging activity, and fraction WRPs-6 with the highest mDP had α-glucosidase and pancreatic lipase inhibitory effects. These findings provide, for the first time, useful information on the structures and potential bioactivities of different WRP fractions. The high proanthocyanidin contents and the potential role of the different WRP fractions in oxidation resistance and management of diabetes and obesity make them worthy of further consideration as functional food ingredients or nutraceuticals. Future in vivo study on these beneficial effects will be carried out to facilitate the applications of WRPs.

## Figures and Tables

**Table 1:** Analysis of variance for the fitted model of BBD.

Source	Sum of Squares	Degree of Freedom	Mean Square	F-Value	*p*-Value
Model	27.15	14	1.94	168.10	<0.0001 *
X_1_	7.74	1	7.74	670.94	<0.0001 *
X_2_	0.13	1	0.13	11.00	0.0051 *
X_3_	0.12	1	0.12	10.00	0.0069 *
X_4_	0.07	1	0.07	5.83	0.0300 *
X_1_X_2_	7.78 × 10^−4^	1	7.78 × 10^−4^	0.067	0.7989
X_1_X_3_	0.35	1	0.35	30.53	<0.0001 *
X_1_X_4_	0.08	1	0.08	6.80	0.0206 *
X_2_X_3_	7.30 × 10^−3^	1	7.30 × 10^−3^	0.63	0.4396
X_2_X_4_	0.27	1	0.27	23.00	0.0003 *
X_3_X_4_	0.14	1	0.14	11.94	0.0039 *
X_1_^2^	12.23	1	12.23	105.89	<0.0001 *
X_2_^2^	9.17	1	9.17	79.41	<0.0001 *
X_3_^2^	0.41	1	0.41	35.66	<0.0001 *
X_4_^2^	0.57	1	0.57	48.97	<0.0001 *
Residual	0.16	14	0.012	-	-
Lack of fit	0.14	10	0.014	2.88	0.1596
Pure error	0.02	4	4.92 × 10^−3^	-	-
Cor total	27.31	28	-	-	-

* Significant difference at *p* < 0.05.

**Table 2:** The proanthocyanidin content, IC_50_ values of DPPH, α-glucosidase, and pancreatic lipase assay of fractions WRPs-1–WRPs-6.

Fraction	Proanthocyanidin Content (mg/g Extract)	IC_50/DPPH_ (μg/mL)	IC_50/α-glucosidase_ (μg/mL)	IC_50/pancreatic lipase_ (μg/mL)
WRPs-1	524.19 ± 3.56 ^e^	74.91 ± 0.83 ^c^	316.07 ± 1.08 ^a^	>2000
WRPs-2	639.92 ± 5.77 ^c^	59.57 ± 1.52 ^d^	304.17 ± 2.46 ^b^	>2000
WRPs-3	863.81 ± 8.02 ^a^	34.29 ± 0.78 ^e^	289.04 ± 3.11 ^c^	>2000
WRPs-4	679.34 ± 4.55 ^b^	36.73 ± 0.96 ^e^	257.20 ± 3.85 ^d^	>2000
WRPs-5	629.16 ± 6.82 ^c^	35.44 ± 1.02 ^e^	117.72 ± 1.45 ^f^	>2000
WRPs-6	567.20 ± 5.76 ^d^	451.85 ± 2.47 ^a^	84.01 ± 0.74 ^g^	1054.01 ± 6.67 ^a^
ascorbic acid	-	100.05 ± 0.94 ^b^	-	-
acarbose	-	-	186.31 ± 1.04 ^e^	-
orlistat	-	-	-	15.45 ± 0.14 ^b^

Data are expressed as mean ± SD (*n* = 3). Values with different letters in the same column are significantly different (*p* < 0.05).

**Table 3:** Cleavage products of wild rice proanthocyanidins after phloroglucinolysis and their major MS data.

Retention Time (min)	[M + H]^+^ (*m*/*z*)			Fragment Ion (*m*/*z*)	Compound
	Measured	Calculated	Error (ppm)		
2.71	431.0970	431.0973	−0.67	305.0655, 263.0478	EGC-Ph
3.35	415.1024	415.1024	−0.06	288.9869, 271.1326, 263.0480	C-Ph
3.67	415.1025	415.1024	0.27	288.9869, 271.1326, 263.0480	EC-Ph
4.18	307.0818	307.0812	1.89	181.0490	EGC
5.30	291.0862	291.0863	−0.56	273.0747, 165.0544	C
6.00	291.0862	291.0863	−0.56	273.0747, 165.0544	EC

Abbreviations: EGC-Ph, (−)-epigallocatechin-phloroglucinol derivative; C-Ph, (+)-catechin-phloroglucinol derivative; EC-Ph, (−)-epicatechin-phloroglucinol derivative; EGC, (−)-epigallocatechin; C, (+)-catechin; EC, (−)-epicatechin.

**Table 4:** Subunit composition (percent in moles) and mean degree of polymerization (mDP) of fractions WRPs-1–WRPs-6.

Fraction	Terminal Unit (%)			Extension Unit (%)			mDP
	C	EC	EGC	C-Ph	EC-Ph	EGC-Ph	
WRPs-1	15.15 ± 0.30 ^a^	11.83 ± 0.42 ^a^	9.55 ± 0.33 ^a^	15.87 ± 0.31 ^d^	44.03 ± 0.53 ^e^	3.57 ± 0.11 ^b^	2.66 ± 0.04 ^f^
WRPs-2	8.95 ± 0.32 ^b^	9.62 ± 0.11 ^b^	4.35 ± 0.05 ^b^	22.89 ± 0.26 ^a^	49.91 ± 0.46 ^d^	3.28 ± 0.09 ^c^	4.32 ± 0.06 ^e^
WRPs-3	9.69 ± 0.47 ^b^	6.55 ± 0.13 ^c^	4.55 ± 0.04 ^b^	20.43 ± 0.24 ^b^	55.62 ± 0.59 ^c^	3.16 ± 0.12 ^cd^	4.81 ± 0.09 ^d^
WRPs-4	7.52 ± 0.48 ^c^	6.60 ± 0.32 ^c^	3.19 ± 0.17 ^c^	16.79 ± 0.45 ^c^	63.03 ± 0.80 ^b^	2.87 ± 0.08 ^e^	5.78 ± 0.12 ^c^
WRPs-5	6.01 ± 0.28 ^d^	4.83 ± 0.09 ^d^	4.45 ± 0.12 ^b^	16.92 ± 0.31 ^c^	64.71 ± 0.65 ^b^	3.08 ± 0.15 ^d^	6.54 ± 0.23 ^b^
WRPs-6	4.21 ± 0.12 ^e^	3.80 ± 0.06 ^e^	1.70 ± 0.04 ^d^	15.79 ± 0.21 ^d^	70.63 ± 0.69 ^a^	3.87 ± 0.10 ^a^	10.30 ± 0.46 ^a^

Data are expressed as mean ± SD (*n* = 3). Abbreviations: C, (+)-catechin; EC, (−)-epicatechin; EGC, (−)-epigallocatechin; C-Ph, (+)-catechin-phloroglucinol derivative; EC-Ph, (−)-epicatechin-phloroglucinol derivative; EGC-Ph, (−)-epigallocatechin-phloroglucinol derivative. Values with different letters in the same column are significantly different (*p* < 0.05).

## Supplementary Materials

The following are available online, Table S1: Box–Behnken design with coded and actual values for the extraction conditions and response values for the content of wild rice proanthocyanidins. Figure S1: Effects of different extraction parameters on the content of wild rice proanthocyanidins in the single factor experiment. Figure S2: Reversed-phase UPLC chromatograms of fractions WRPs-1–WRPs-6 eluted from Sephadex LH-20 column at 280 nm.
