# Supplementary material for: Extraction of Proanthocyanidins from Chinese Wild Rice (Zizania latifolia) and Analyses of Structural Composition and Potential Bioactivities of Different Fractions
Source: Molecules. 2019 Apr 30;24(9):1681. doi: 10.3390/molecules24091681 (PMC6539017; doi:10.3390/molecules24091681)
Supplement: Supplementary file 1 [file molecules-24-01681-s001.pdf]

Table S1. Box–Behnken design with coded and actual values for the extraction conditions and response values for the content of wild rice proanthocyanidins (WRPs).

Run	Extraction Variables				WRPs Content (mg/g rice)	
	X_1	X_2	X_3	X_4	Experimental	Predicted
1	1 (100)	0 (50)	1 (50)	0 (350)	4.90	4.87
2	0 (90)	1 (60)	0 (40)	1 (400)	4.08	4.17
3	0 (90)	0 (50)	1 (50)	1 (400)	5.57	5.55
4	0 (90)	0 (50)	0 (50)	0 (350)	6.15	6.09
5	0 (90)	−1 (40)	0 (40)	1 (400)	5.05	4.89
6	0 (90)	1 (60)	1 (50)	0 (350)	4.40	4.45
7	0 (90)	0 (50)	1 (50)	−1 (300)	5.36	5.33
8	0 (90)	−1 (40)	1 (50)	0 (350)	4.67	4.52
9	−1 (80)	1 (60)	0 (40)	0 (350)	2.77	2.62
10	−1 (80)	0 (50)	1 (50)	0 (350)	3.82	3.86
11	1 (100)	0 (50)	−1 (30)	0 (350)	5.76	5.66
12	0 (90)	1 (60)	0 (40)	−1 (300)	4.74	4.83
13	1 (100)	0 (50)	0 (40)	−1 (300)	5.39	5.44
14	0 (90)	−1 (40)	0 (40)	−1 (300)	4.67	4.65
15	0 (90)	0 (50)	0 (40)	0 (350)	6.09	6.09
16	0 (90)	−1 (40)	−1 (30)	0 (350)	4.75	4.85
17	0 (90)	0 (50)	0 (40)	0 (350)	5.97	6.09
18	0 (90)	0 (50)	0 (40)	0 (350)	6.10	6.09
19	−1 (80)	0 (50)	0 (40)	1 (400)	3.67	3.68
20	1 (100)	0 (50)	0 (40)	1 (400)	4.97	5.01
21	0 (90)	0 (50)	0 (40)	0 (350)	6.13	6.09
22	0 (90)	0 (50)	−1 (30)	−1 (300)	5.88	5.90
23	0 (90)	1 (60)	−1 (30)	0 (350)	4.65	4.64
24	−1 (80)	−1 (40)	0 (40)	0 (350)	2.72	2.83
25	1 (100)	−1 (40)	0 (40)	0 (350)	4.31	4.43
26	−1 (80)	0 (50)	−1 (30)	0 (350)	3.49	3.46
27	0 (90)	0 (50)	−1 (30)	1 (400)	5.35	5.38
28	−1 (80)	0 (50)	0 (40)	−1 (300)	3.53	3.55
29	1 (100)	1 (60)	0 (40)	0 (350)	4.30	4.23

X_{1-4}: composition of aqueous ethanol (EtOH %, *v*/*v*), liquid-solid ratio (mL/g, *v*/*w*), extraction temperature (℃), and ultrasonic power (W).

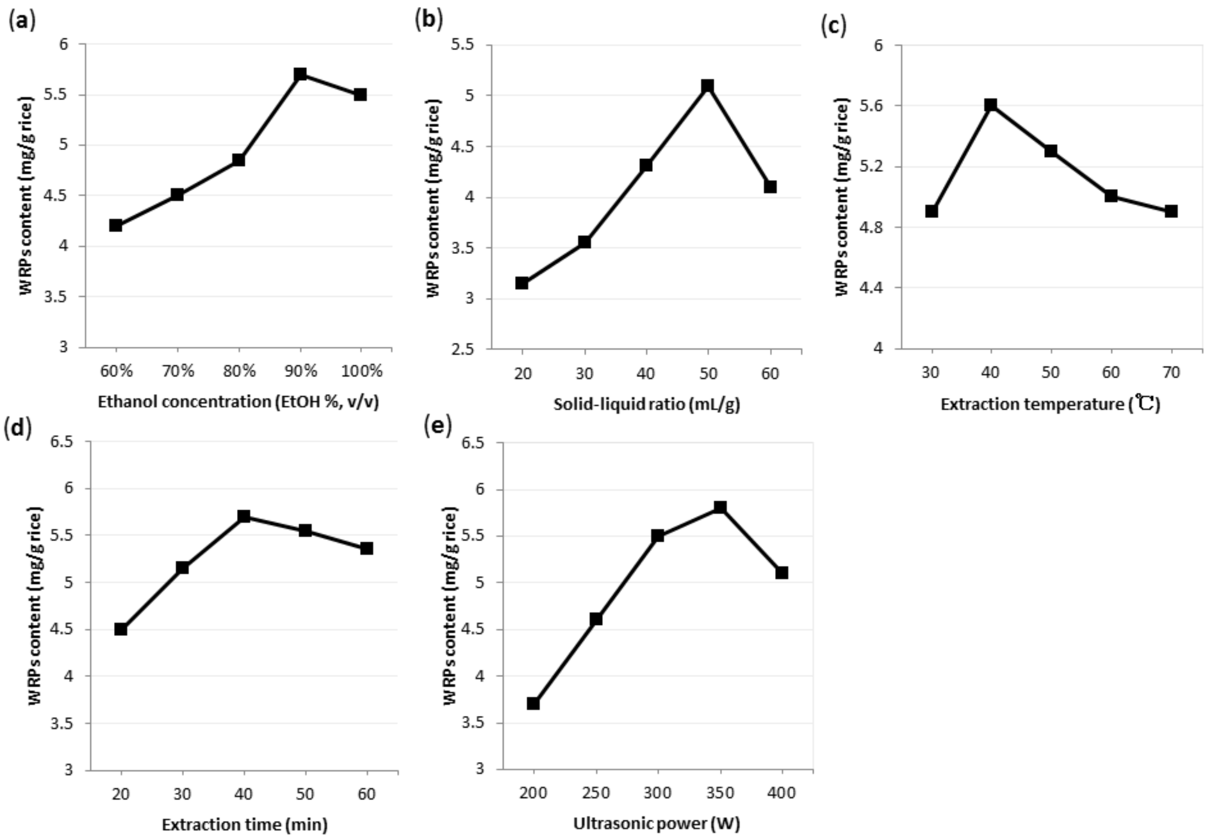

Figure S1. Effects of different extraction parameters on the content of wild rice proanthocyanidins in the single factor experiment.

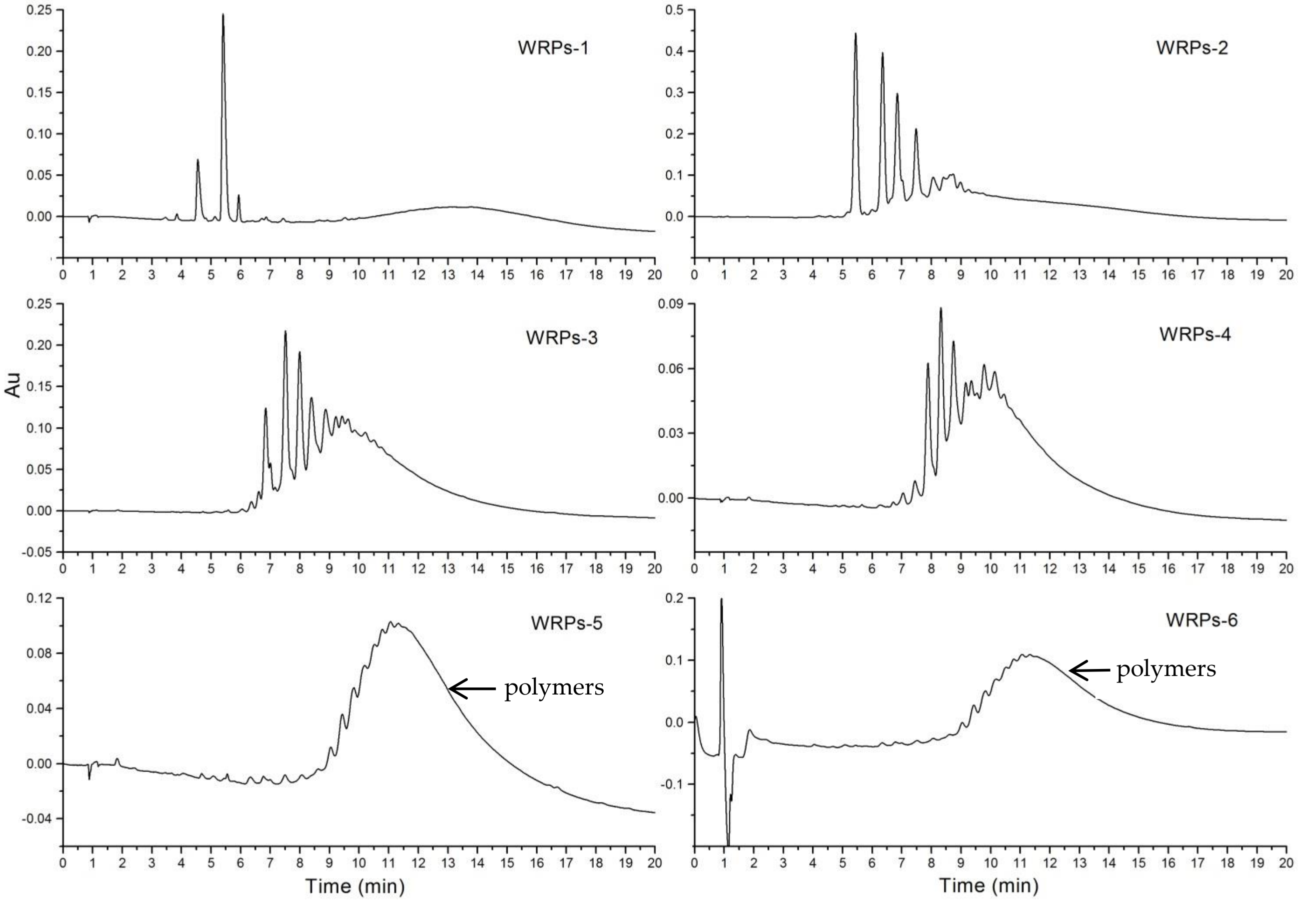

Figure S2. Reversed-phase UPLC chromatograms of fractions WRPs-1−WRPs-6 eluted from Sephadex LH-20 column at 280 nm. Some proanthocyanidin polymers could not be resolved but were co-eluted as a large unresolved peak. The gradient solvent system consisting of A (acetonitrile containing 0.1% acetic acid, *v*/*v*) and B (water containing 0.1% acetic acid, *v*/*v*) was as follows: 0–5 min, 5–7% A; 5–10 min, 7–10% A; 10–15 min, 10–20% A; 15–18 min, 20–90% A; 18–20 min, 90–5% A.
